# Interprofessional collaboration in Primary Health Care: preceptors and professors in Education Program for Health Work

**DOI:** 10.1590/1980-220X-REEUSP-2024-0206en

**Published:** 2025-09-08

**Authors:** Elaine Andrade Leal Silva, Rosana Maria de Oliveira Silva, Ana Lúcia Arcanjo Oliveira Cordeiro, Queuam Ferreira Silva de Oliveira, Rafaela Braga Pereira Velôso, Gilberto Tadeu Reis da Silva

**Affiliations:** 1 Universidade Federal do Recôncavo, Centro de Ciências da Saúde, Santo Antônio de Jesus, BA, Brazil.; 2 Universidade Federal da Bahia, Escola de Enfermagem, Salvador, BA, Brazil.; 3 Universidade do Estado da Bahia, Departamento de Educação, Senhor do Bonfim, BA, Brazil.; 4 Universidade Estadual de Feira de Santana, Departamento de Saúde, Feira de Santana, BA, Brazil.

**Keywords:** Interprofessional Education, Patient Care Team, Preceptorship, Health, Primary Health Care

## Abstract

**Objective:**

This article aims to analyze the performance of professors and preceptors of the Education Program for Health Work in the development of interprofessional collaboration in Primary Health Care.

**Method:**

Descriptive study, with a qualitative approach, in which professors and preceptors from the Education Program for Health Work of five federal universities in the Brazilian Northeast participated. Between September and October 2020, 44 workers responded to the online questionnaire. Data were processed according to analytical procedures described by Bardin and interpreted in light of the four-dimensional Model of Interprofessional Collaboration with the aid of webQDA® software.

**Results:**

Three categories were identified: 1) Provision for sharing common goals; 2) Mobilization for internalization of interdependence between professionals, and 3) Monitoring and adjustment for the development of interprofessional collaboration.

**Conclusion:**

The study revealed that the role of professors and preceptors in developing interprofessional collaboration is significantly focused on the interactive conduct between health professionals and students, as well as on the development of actions and adjustments for collaborative work.

## INTRODUCTION

Interprofessional collaboration in Primary Health Care (PHC) is an essential element for qualifying health care, as it encourages the integration of knowledge and practices among different professionals. Based on Interprofessional Education (IPE) and interprofessional practice, this approach becomes a strategic resource to strengthen teamwork and enhance health outcomes, especially when facing challenges such as chronic conditions and social vulnerability.

Defined as a collective action, Interprofessional Collaboration (IPC) is based on the partnership between health professionals and users, on sharing (planning, intervention and responsibilities), on interdependence (from one to the other), and on power (shared between team members)^(1)^. The adoption of strategies that encourage this collaboration, such as education through work and the implementation of active teaching-learning methodologies, contributes significantly to the transformation of practices and the qualification of care in PHC^(2)^.

IPC takes place in health and community service contexts^(2,3)^ and, according to the literature, there are efforts by educational institutions in interprofessional education programs, reflections on successful experiences in the context of PHC, with the aim of preparing future health professionals for the development of interprofessional practice^(4,5)^. A study carried out in Singapore with doctors and nurses concluded that IPC improves the results of people assisted, as well as the satisfaction of professionals and health costs^(6)^. Research in Europe and North America advances the propagation of innovative health training processes focused on the users’ health needs^(7,8)^.

In Brazil, research announces restricted institutional support^(9,10)^ for interprofessional teaching and practice, team interventions^(11,12)^, flexibility in the curriculum, the inclusion of interprofessional experiences^(7,9)^, the teaching-service articulation^(9)^, and professional performance that moves away from mechanized and fragmented practice^(10)^. Other studies, based on actions developed in the Education Program for Health Work (*PET-Saúde*), highlight the development of collaborative activities, in which students, professors and health professionals, together, in an interactive and interdependent way, shared learning and exchanged knowledge between different courses and occupations^(11,13)^.

However, in practice, there is still fragmentation of health care, in addition to distorted perceptions about professions^(12)^. Investing in the development of students and healthcare professionals^(4)^, as well as in welcoming, in teamwork^(5)^, in more collaborative and community-based health care practices^(10,12)^, can contribute to the reorientation of training and performance in health.

As proposed in the aforementioned program, the participating universities develop their actions based on the theoretical and methodological bases of IPE^(14,15)^ with the aim of promoting interprofessionality and training professionals capable of collaborative work. That said, *PET-Saúde*/Interprofessionality is considered a space for interaction among health professionals that encourages curricular changes aligned with the curricular guidelines of health courses and induces multi- and interprofessional work in teaching-service settings^(16)^. However, obstacles to the advancement of IPE persist in Brazilian universities^(10)^, which requires continued investment in health training and the mobilization of research into the performance of educators in conducting IPC in *PET-Saúde*/Interprofessionality.

The importance of the leading role of professors and preceptors in the planning, management, and execution of the IPC developed in the Program is highlighted, as well as in the dimensions of the four-dimensional model developed by D’Amour and contributors^(17)^. Knowing how the training process has been developed in IPE-inducing programs, based on a model that analyzes relational and organizational dimensions, can contribute to the evaluation and implementation of the Program and interprofessional curricula.

This model is structured in four dimensions: common objectives and shared vision, internalization, formalization, and governance. These dimensions interact and influence each other. All of them support the IPC^(17)^.

The dimensions “Common objectives” and “Shared vision” are related to the interaction between professionals and the distribution of responsibilities. The “Internalization” dimension is related to the awareness of interdependence with other professionals and the nature of their work. In “Formalization”, rules are established and related to clinical interventions and interaction modalities are instituted. And finally, the “Governance” dimension concerns organizations and their external and internal factors of structure and power^(17)^.

Therefore, the following objective was defined: to analyze the performance of professors and preceptors of the Education Program for Health Work in the development of interprofessional collaboration in Primary Health Care.

## METHOD

### Design of Study

Descriptive research, of a qualitative nature, which is part of the thesis entitled “Colaboração Interprofissional no Programa de Educação pelo Trabalho para a Saúde/Interprofissionalidade (Interprofessional Collaboration in the Education Program for Health Work/Interprofessionality”. The checklist of the *Consolidated Criteria for Reporting Qualitative Research* (COREQ)^(18)^ was applied and the requirements related to the methods and other topics, which will be discussed below, were followed.

### Local

Five federal universities that develop *PET-Saúde*/Interprofessionality activities in PHC in Northeast Brazil, which participated in the program in two previous editions (public notice no. 14/2013 – *PET-Saúde*/Health Care Networks and public notice no. 13/2015 PET-Saúde/Gradua SUS) in the same *campus*.

### Population

PET/Interprofessionality professors and preceptors. The inclusion criterion established was to be active in the Program for a period of more than 12 months. A total of 130 people were invited by email (invitation letter), including PET/Interprofessionality professors and preceptors. Of these, 60 accepted the invitation and 44 responded to the forms, resulting in a response rate of 73%. For those who accepted the invitation, the Free and Informed Consent Form (FICF) and the access link to the questionnaire were sent. A period of 30 days was established as the average time between sending the invitation and receiving the online responses.

### Data Collection

In the data collection phase, six pilot tests were carried out with preceptors and professors from *PET-Saúde*/Interprofessionality at state universities, following this protocol: invitation, sending of the FICF with questionnaire access link, receipt and analysis of responses, with final comments.

After improving the collection instrument, data collection was carried out using an open questionnaire online, with instructions on how to fill it out, containing 14 questions, prepared and applied by an experienced researcher, through the access link made available via *Google Forms*® between the months of August and October 2020. The time to complete the questionnaire was 10 to 12 minutes.

Of the questions formulated, those related to the characterization of the participants (sex, degree) and responses regarding the strategies used for introduction and adjustments to the IPC were selected. There was no refusal or suspension of participation in the research. All participants received a copy of the responses, which allowed them to confirm them.

### Data Analysis and Treatment

Data from the questionnaire via *Google Forms*® were transported to an Excel spreadsheet and transposed as internal sources to the software webQDA®. With the help of this software, the researchers built the *corpus* of the research, carried out the data coding and analysis operations, and established the indexes and absolute frequency of the 50 most recurring words related to IPC in *PET-Saúde*/Interprofessionality.

The coding tree adopted a hierarchical format, based on the classification of sources according to criteria named a priori as: identification and sharing of common objectives, internalization of the interdependence of another health professional, and adjustment for the development of the IPC.

Data analysis and processing were carried out using content analysis^(19)^, anchored in the theoretical framework of the IPC Structuring Model^(17)^, which is based on the principle of IPC in Primary Health Care contexts and is present in the process of emergence and structuring, from the perspective of interactions.

Data categorization was done by aggregating the recording units using the theme-word criterion, that is, based on the thematic statement. Then, inferences were made from the data.

### Ethical Aspects

The research was approved on July 1, 2020 by the Research Ethics Committee (CEP) of the Nursing School of the Universidade Federal da Bahia, with opinion number 4.127.223. Ethical commitments were respected, in accordance with the guidelines of the National Health Council, for procedures in research with human beings, at any stage and in a virtual environment^(20)^. To ensure the interviewees’ anonymity, the codename Interviewee was used with the letter E, followed by the ordinal number presented in ascending order, such as E1, E2 and so on. As recommended by Open Science, the link of the repository in which the research data were deposited was made available.

## RESULTS

The study was carried out with 44 participants (26 preceptors, 18 PET professors), the majority of whom were female, with different degrees in health, such as Biological Sciences, Nursing, Physiotherapy, Medicine, Nutrition, Dentistry, Psychology, and Social Work. Of these, the largest percentage (45.5%) was from Nursing.

For better understanding, and in light of the IPC Structuring Model^(17)^, the performance of professors and preceptors in conducting the IPC is described in three of the four dimensions expressed in the results, in the following established categories: 1) Provision for sharing common goals; 2) Mobilization for the internalization of interdependence among professionals, and 3) Monitoring and adjustment for the development of interprofessional collaboration.

1) Provision for Sharing Common Goals:

During the activities carried out at *PET-Saúde*/Interprofessionality, professors and preceptors guided the insertion of students into the health service and the work process of the health team.

*The insertion into the reality of the service and the work process of the health team, based on the planning, execution and discussion of the actions carried out inside and outside the Primary Care Health Unit* [...] *in an articulated manner with the services and management* (E6).

[...] *Through the Situational Diagnosis raised, it was possible to investigate possible problems and obstacles to be worked on to improve assistance* (E12).

Among the actions developed, team meetings should be highlighted, which encouraged interaction and the establishment of common objectives. At the same time, there was encouragement to listen to other health professionals, as well as to users and the community, as indicated below:

*Bring together all members of the teams in which the tutorial groups work, hold workshops, meetings, debates to gain knowledge about the professions and practices and to identify common points among the occupations, plan activities from an interprofessional perspective, encourage listening to users and reflection on the service’s response to users’ demands* [...] (E5).

*Meetings to define priorities, planning and programming, evaluation, creation of activity agendas, organization and implementation of activities, thematic studies, all done jointly* (E39).

The participants’ responses revealed that the interaction of the Program’s participants in small groups and conceptual alignment constitute other actions undertaken by the *PET-Saúde*/Interprofessionality advisors. These actions took place in the pandemic context experienced at the time:

*During this period* [...] *we have invested in the qualification of students, tutors and professors,* [...]. *Meetings and gatherings continue to take place remotely* (E6).

[...] *we seek to maintain the organization of activities in small groups* (preserving the multidisciplinary composition) *and regular meetings with all participants of the Tutorial Learning Group to share experiences and discuss conceptual aspects.* […] (E7)

2) Mobilization for the Internalization of interdependence among professionals:

In this category, it was observed that actions related to the internalization of the interdependence of others start from debates on the attributions of each occupation, discussion of cases, articulation centered on the community and development of the mastery of collaborative skills, as described below:

*Debates that promote knowledge of the role of each profession, discussion of problematic cases/situations to identify the role of each profession in solving the problem, with discussion of specific, common and collaborative skills* [...] (E7).

*The pedagogical design of the PET-Saúde/Interprofessionality actions in which I participate had two principles as its intention: community-centered care and focus on the individual and four domains of competences: interprofessional communication, professional roles, teamwork, ethics, and interprofessional values* (E18).

Moreover, the interviewees demonstrated that they use joint action, which can occur during the implementation of a therapeutic project, home visits, or the execution of a conversation circle.

*The activities are developed in the Primary Care Health Units, together with the team’s professionals and the community* [...] *Permanent education, unique therapeutic project, consultations and shared home visits* [...] (E8).

[...] *division of work groups, respecting the diversity of courses and professionals, monitoring of the execution of activities, discussion groups with members of different professions* [...] (E26).

According to some participants, the actions carried out jointly result in a chain of activities established to prioritize the need for interdependence between Program participants, users and staff:

*With the team, the focus was on developing strategies for collaborative work; with users, the focus was on identifying health care demands to strengthen interprofessional actions* [...] (E11).

[...] *The activities to be developed with each family and the goal to be achieved are outlined. Activities are developed, evaluated, and applied. It is then taken to a group discussion* (students, tutors and professors) *and the success or failure of the activity/service is evaluated. This way, families are served in an interprofessional manner. To solve problems present in these families* [...] (E25).

In one of the interviews, this partnership was mentioned by a *PET-Saúde*/Interprofessionality participant:

*Participation of students, professionals, and the community in the daily practices of the Service, with the opportunity for everyone to participate in health care processes* [...] (E12).

[...] *partnership with organized patient networks, meetings with patients and their attending physicians, individual and group studies, research on care provided to service users via suggestion boxes* (E13).

3) Monitoring and adjustment for the development of interprofessional collaboration:

The third category is related to the adjustments made by *PET-Saúde*/Interprofessionality professors and preceptors for the development of the IPC. One of the actions was the (re)conduction of collaborative work, through dialogue and feedback from the community:

*Dialogue is always the first step. Weekly groups and meetings greatly assist in organizing activities and aligning care actions and interaction and intervention strategies* (E22).

*One of the community’s demands was the implementation of the women’s group. With each action carried out, feedback was collected from the participating community itself, meaning that the next actions and participations could be planned and adjusted* [...] (E37).

Another aspect arising from the statements concerns the alignment of conduct and redefinition of activity routes:

*Study of scientific articles on Interprofessionality, recognition of the multidisciplinary team, and analysis of the expanded consultation* [...]*. To build unique therapeutic projects (PTS) to align conduct and referrals* (E3).

*We have encouraged debate on how teamwork has been carried out, how members have been listened to when carrying out tasks, what strategies they have used to resolve conflicts between members and when making collective decisions* [...] *to encourage reflection and the adoption of supportive and collaborative practices* (E44).

Regarding adjustments for the development of the IPC, the findings show intra- and inter-institutional mobilization in some health and education sectors:

[...] *We involved the* (human resources sector) *of the Municipal Health Department in all stages of the project, keeping them aware of all planning and activities carried out; professors discuss the topic of interprofessionality in meetings of the Structuring Teaching Center* [...] (E5).

[...] *Holding meetings with health course departments, seeking to raise awareness among the various training stakeholders* (E41).

## DISCUSSION

The representativeness of nurses is notable in terms of the performance of professors and preceptors of the Education Program for Health Work in the development of IPC in PHC, consistent with the representativeness of the profession in different scenarios. Authors argue that nurses have more positive attitudes towards interprofessional collaboration^(6)^.

Acting as professors or preceptors together with other professional categories, nurses mobilize actions to share common objectives, internalization of interdependence among health professionals, and monitoring and adjustments for the development of the IPC. Knowledge about the strategies used by nurses and other health professionals highlights the importance of investing in learning experiences and interprofessional care, training focused on experimentation, immersion, and reflection in contexts of teamwork in family and community health care.

Regarding the sharing of common goals - this being the starting point towards the IPC, by inserting students into the daily routine and work processes of the family health team in an interactive manner and in small groups, the facilitators direct their actions in convergence with the IPC Structuring Model^(17)^. By promoting listening within professionals, users, and the community, students are encouraged to make shared decisions. Thus, they seek to produce responses to the reality encountered, as verified in other studies developed in similar contexts^(13,21)^. However, although teamwork is a reality in PHC^(22)^, the valuation of individual consultation is still perceived^(23)^.

Regarding the mobilization for the internalization of interdependence between health professionals, this study showed that *PET-Saúde*/Interprofessionality professors and preceptors are willing to invest in mutual coexistence and in a relationship of trust, important elements in the dimension of the Internalization of IPC. The debates, case discussions, consultations, and shared visits among *PET-Saúd*e/Interprofessionality participants allow knowing about the other health professional and influence the dimensions of “Common objectives” and “Shared goals”, as they get to know about collaborative care and capture the knowledge of the other.

When students are encouraged to create therapeutic projects, seek articulation with patients and their organizational network, the development of interprofessional communication and care centered on the person, family and community is enhanced^(8)^. Developing such skills is essential for the development of interprofessional collaboration^(24,25)^.

Although the potential of improving healthcare through user-centered care is evident, it is still a challenge to involve users in decision-making about their healthcare, as recommended by the World Health Organization^(26,27)^.

In monitoring and adjusting the development of the IPC, it is connected to two dimensions of the IPC Structuring Model: Formalization and Governance^(17)^. The alignment of conduct and the redefinition of activity routes conducted by professors and preceptors in this study are aligned with the “Formalization” dimension, since they are presented in the form of information exchange and formatting of the work process.

The dialogue between the participants of *PET-Saúde*/Interprofessionality and the feedback of the community in the re(conduction) of collaborative work allows valuing and respecting the user, clarifying responsibilities, and negotiating how they will be shared. Consequently, it is essential to overcome barriers and develop clear communication^(10)^, since, according to the research, the compromise of synergy among the team and the difficulty in communication constitute obstacles to interprofessional collaboration^(4)^.

Another action identified for monitoring and adjusting the development of the IPC was intra- and inter-institutional mobilization. This action is in line with the “Governance” dimension of the IPC Structuring Model. The strategic political role of PET professors and preceptors provided clear direction for students, as well as guidance for collaborative learning.

In the United States of America, a teaching-service integration program similar to *PET-Saúde*/Interprofessionality invested in intra- and inter-institutional mobilization to maintain IPC in health services^(8)^. In addition to the Brazilian experience of *PET-Saúde*/Interprofessionality, other countries such as South Africa^(21)^ and Australia^(28)^ invested in the creation of interprofessional disciplines and internships as spaces for the development of IPC.

Interprofessional learning experiences focused on internalizing the interdependence of others prove to be significant as they stimulate change, both in the training and in the performance of health professionals. This type of learning is in line with andragogy, adult education, and contributes to the development of skills aimed at decision-making^(29)^.

The limitations of this study lie in the lack of in-depth analysis of issues related to the influence of exogenous and structural factors, which interfere in the individuals’ relational processes. Thus, investigative proposals on other determinants of the collaborative process are required.

Advances in the area of health and nursing are also related to the continued mobilization that *PET-Saúde*/Interprofessionality has to develop clinical practices based on collaboration and interaction among professionals, which allow better integration for health practices and contribute to paradigm changes to overcome the fragmentation of care and the supremacy of knowledge.

## CONCLUSION

The results of the study allowed us to identify the guided actions carried out in *PET-Saúde*/Interprofessionality, from the perspective of professors and preceptors. The work of professors and preceptors in the Program favored approaches to interprofessional collaboration, by enabling students to introduce new practices in the relational sphere and the structuring of collective action between the university and health services. The study also revealed that facilitators are willing to conduct an interprofessional educational project. However, their work is hampered by the informality of the collaborative work process, the low governability of collaborative actions, and the weak cohesion between the university and the health service, towards the implementation of interprofessional collaboration in health.

Given the contributions and limitations, there is a need to invest in the sharing of knowledge among those involved, respecting freedom of decision and involving families and the community. In addition, further research assessing collaboration in the effectiveness of behavioral changes among health professionals, in different spaces of integration among teaching, service, and the community is required.

## Data Availability

The entire dataset supporting the study’s results was published in the article itself.

## References

[1] D’Amour D, Ferrada-Videla M, San Martin Rodriguez L, Beaulieu M-D. (2005). The conceptual basis for interprofessional collaboration: core concepts and theoretical frameworks. J Interprof Care.

[2] Organização Mundial de Saúde (2010). Marco para ação em educação interprofissional e prática colaborativa [Internet].

[3] World Health Organization (2010). Framework for action on interprofessional education and collaborative practice [Internet]. https://www.who.int/hrh/resources/framework_action/en/.

[4] Fonseca CL, Costa LA, Freitas BHBM, Alencasteo LCS, Mizoguchi MV (2021). Cuidado interprofissional à criança e ao adolescente: revisão de escopo. Rev Conex UEPG.

[5] Queiroz DM, Oliveira LC, Araújo Fo PA, Silva MRF (2021). Challenges and potentials of the production of comprehensive care in Primary Health Care in Brazil. Rev Bras Enferm.

[6] Zheng RM, Sim YF, Koh GC-H. (2016). Attitudes towards interprofessional collaboration among primary care physicians and nurses in Singapore. J Interprof Care.

[7] Sunguya BF, Hinthong W, Jimba M, Yasuoka J. (2014). Interprofessional education for whom? Challenges and lessons learned from its implementation in developed countries and their application to developing countries: a systematic review. PLoS One.

[8] Banister G, Portney LG, Vega-barachowitz C, Jampel A, Schnider ME, Inzana R (2019). The interprofessional dedicated education unit: design, implementation and evaluation of an innovative model for fostering interprofessional collaborative practice. J Interprof Educ Pract.

[9] Silva JAM, Peduzzi M, Orchard C, Leonello VM (2015). Educação interprofissional e prática colaborativa na Atenção Primária à Saúde. Rev Esc Enferm USP.

[10] Tonin CF, Souza JM, Manfrini GC, Heidemann ITSB, Durand MK (2021). Arakawa-Belaunde AM. How’s mental health? Dialogues and reflections on care strategies in Primary Health Care Care. Strategies in APS in Mental Health. Res Soc Dev.

[11] Busse ACS, Ferreira FG, Mendes GF, Evangelista RA, Matos SQS, Anjos WBA (2021). The interface between Education through Work for Health Program/Interprofessionality and the National Policy of Permanent Education in Health. Rev Ibero-Americana Humanidades. Ciênc Educ.

[12] Villela EFM, Diniz TM, Ferreira BR, Rocha MGS, Garcia LPRR, Zanuzzi TRL (2021). O papel da educação interprofissional no processo de reorientação da formação em saúde. NTQR.

[13] Brasil, Ministério da Saúde, Secretaria de Gestão do Trabalho e da Educação na Saúde (2018). Edital nº 10, 23 de Julho de 2018. Seleção para o Programa de Educação pelo Trabalho para a Saúde PET-Saúde/Interprofissionalidade.

[14] Barr H. (1998). Towards a competency-based model for interprofessional education. J Interprof Care.

[15] Silva EAL, Silva RMO, Cordeiro ALAO, Silva GTR, Velôso RBP, Silva MES (2023). Interprofessional collaboration in the program for education at work for health. Cienc Cuid Saude.

[16] França T, Magnago C, Santos MR, Belisário SA, Silva CBG (2018). PET-Saúde/GraduaSUS: retrospectiva, diferenciais e panorama de distribuição dos projetos. Saúde Debate.

[17] D’Amour D, Goulet L, Labadie J-F, Martín-Rodriguez LS, Pineault R. (2008). A model and typology of collaboration between professionals in healthcare organizations. BMC Health Serv Res.

[18] Santos Souza VR, Marziale MHP, Silva GTR, Nascimento PL (2021). Translation and validation into Brazilian Portuguese and assessment of the COREQ checklist. Acta Paul Enferm.

[19] Bardin L. (2016). Análise de conteúdo.

[20] Brasil, Ministério da Saúde (2021). Orientações para procedimentos em pesquisas com qualquer etapa em ambiente virtual, de 24 de fevereiro de 2021 [Internet].

[21] Snyman S, Geldenhuys M. (2019). Exposing an interprofessional class of first years to an underserved community contributed to students’ contextualisation of the determinants of health. J Interprof Care.

[22] Peduzzi M, Agreli HF (2018). Teamwork and collaborative practice in primary health care. Interface Commun Heal Educ.

[23] Monteiro PN, Pícoli RP, Souza GRM (2021). Scope of practices of the Extended Family Health Center (NASF): perspective of professionals from NASF and the Family. Brazilian J Dev.

[24] Canadian Interprofessional Health Collaborative (2010). A national interprofessional competency framework [Internet].

[25] Barr H, Low H. (2013). Introdução à Educação Interprofissional [Internet].

[26] World Health Organization, United Nations Children’s Fund (2018). A vision for health care in the 21st Century [Internet].

[27] Silva IS, Arantes CIS, Fortuna CM (2019). Conflict as a possible catalyst for democratic relations in the work of the Family Health team. Rev Esc Enferm USP.

[28] Brewer ML, Flavell HL, Jordon J. (2017). Interprofessional team-based placements: the importance of space, place, and facilitation. J Interprof Care.

[29] Oandasan I, Reeves S. (2005). Key elements for interprofessional education. Part 1: the learner, the educator and the learning context. J Interprof Care.

