# Supplementary material for: Interprofessional collaboration in Primary Health Care: preceptors and professors in Education Program for Health Work
Source: Rev Esc Enferm USP. 2025 Sep 8;59:e20240206. doi: 10.1590/1980-220X-REEUSP-2024-0206en (PMC12448238; doi:10.1590/1980-220X-REEUSP-2024-0206en)
Supplement: Translation

# Translation: Colaboração interprofissional na Atenção Primária à Saúde: preceptores e docentes na Educação pelo Trabalho em Saúde

**DOI:** 10.1590/1980-220X-REEUSP-2024-0206pt

**Authors:** Elaine Andrade Leal Silva, Rosana Maria de Oliveira Silva, Ana Lúcia Arcanjo Oliveira Cordeiro, Queuam Ferreira Silva de Oliveira, Rafaela Braga Pereira Velôso, Gilberto Tadeu Reis da Silva

**Affiliations:** 1 Universidade Federal do Recôncavo, Centro de Ciências da Saúde, Santo Antônio de Jesus, BA, Brasil.; 2 Universidade Federal da Bahia, Escola de Enfermagem, Salvador, BA, Brasil.; 3 Universidade do Estado da Bahia, Departamento de Educação, Senhor do Bonfim, BA, Brasil.; 4 Universidade Estadual de Feira de Santana, Departamento de Saúde, Feira de Santana, BA, Brasil.

**Keywords:** Educação Interprofissional, Equipe de Assistência ao Paciente, Preceptoria, Saúde, Atenção Primária à Saúde

## Abstract

**Objetivo:**

O presente artigo objetiva analisar a atuação de docentes e preceptores do Programa de Educação pelo Trabalho para a Saúde no desenvolvimento da colaboração interprofissional na Atenção Primária à Saúde.

**Método:**

Estudo descritivo, de abordagem qualitativa, do qual participaram docentes e preceptores do Programa de Educação pelo Trabalho para a Saúde de cinco universidades federais do Nordeste brasileiro. Entre setembro e outubro de 2020, 44 trabalhadores responderam ao questionário on-line. Os dados foram tratados conforme procedimentos analíticos descritos por Bardin e interpretados à luz do Modelo quadridimensional da Colaboração Interprofissional com auxílio do software webQDA®.

**Resultados:**

Foram identificadas três categorias: 1) Condução para o compartilhamento de objetivos comuns; 2) Mobilização para a internalização da interdependência entre profissionais e 3) Acompanhamento e ajuste para o desenvolvimento da colaboração interprofissional.

**Conclusão:**

O estudo revelou que a atuação de docentes e preceptores para o desenvolvimento da colaboração interprofissional está centrada de modo relevante na conduta interativa entre profissionais de saúde e estudantes, bem como no desenvolvimento de ações e ajustes para o fazer colaborativo.

## INTRODUÇÃO

A colaboração interprofissional na Atenção Primária à Saúde (APS) é um elemento essencial para a qualificação do cuidado em saúde, uma vez que incentiva a integração de saberes e práticas entre diferentes profissionais. Fundamentada na Educação Interprofissional (EIP) e na prática interprofissional, essa abordagem torna-se um recurso estratégico para fortalecer o trabalho em equipe e potencializar os resultados em saúde, especialmente no enfrentamento de desafios como as condições crônicas e a vulnerabilidade social.

Definida como uma ação coletiva, a Colaboração Interprofissional (CIP) está pautada na parceria entre profissionais de saúde e usuários, no compartilhamento (planejamento, intervenção e responsabilidades), na interdependência (de um para com o outro) e no poder (compartilhado entre integrantes da equipe)^(1)^. A adoção de estratégias que incentivem essa colaboração, como a educação pelo trabalho e a implementação de metodologias ativas de ensino-aprendizagem, contribui significativamente para a transformação das práticas e a qualificação da assistência na APS^(2)^.

A CIP acontece em contextos dos serviços de saúde e comunitários^(2,3)^ e, conforme a literatura, existem esforços das instituições de ensino em programas de educação interprofissional, reflexões frente às experiências exitosas no contexto da APS, com o intuito de preparar os futuros profissionais de saúde para o desenvolvimento da prática interprofissional^(4,5)^. Estudo realizado em Singapura com médicos e enfermeiros concluiu que a CIP melhora os resultados das pessoas assistidas, bem como a satisfação dos profissionais e os custos em saúde^(6)^. Pesquisas na Europa e América do Norte avançam na propagação de processos inovadores de formação em saúde voltados às necessidades de saúde do usuário^(7,8)^.

No Brasil, pesquisas anunciam o apoio institucional restrito^(9,10)^ para o ensino e a prática interprofissional, as intervenções em equipe^(11,12)^, a flexibilidade no currículo, a inclusão de experiências interprofissionais^(7,9)^, a articulação ensino-serviço^(9)^ e a atuação profissional que se distancie da prática mecanizada e fragmentada^(10)^. Outras pesquisas, a partir das ações desenvolvidas no Programa de Educação pelo Trabalho para saúde (PET-Saúde), destacam o desenvolvimento de atividades colaborativas, nas quais estudantes, docentes e profissionais de saúde, juntos, de modo interativo e interdependente, compartilharam aprendizagens, trocaram saberes entre diferentes cursos e profissões^(11,13)^.

No entanto, na prática, ainda existe a fragmentação do cuidado em saúde, além de percepções distorcidas sobre as profissões^(12)^. Investir no desenvolvimento de estudantes e profissionais de saúde^(4)^, bem como no acolhimento, no trabalho em equipe^(5)^, nas práticas de cuidado em saúde mais colaborativas e de base comunitária^(10,12)^, pode contribuir para a reorientação da formação e da atuação na saúde.

Como proposto no programa mencionado, as universidades participantes desenvolvem suas ações fundamentadas nas bases teóricas e metodológicas da EIP^(14,15)^ com o intuito de fomentar a interprofissionalidade, assim como formar profissionais aptos para o trabalho colaborativo. Isto posto, considera-se o PET-Saúde/Interprofissionalidade um espaço de interação entre profissionais de saúde que estimula mudanças curriculares alinhadas às diretrizes curriculares dos cursos de saúde e induz o trabalho multi e interprofissional nos cenários de ensino-serviço^(16)^. No entanto, obstáculos para o avanço da EIP persistem nas universidades brasileiras^(10)^, o que requer a continuidade de investimento na formação em saúde e a mobilização da investigação sobre a atuação de educadores na condução da CIP no PET-Saúde/Interprofissionalidade.

Ressalta-se a importância do protagonismo de professores e preceptores no planejamento, condução e execução da CIP desenvolvida no Programa, bem como nas dimensões do modelo quadridimensional elaborado por D’Amour e colaboradores^(17)^. Conhecer como o processo formativo vem sendo desenvolvido em programas indutores da EIP, pautado em um modelo que analisa as dimensões relacionais e organizativas, pode contribuir para a avaliação e implementação do Programa e de currículos interprofissionais.

Esse modelo estrutura-se em quatro dimensões: objetivos comuns e visão compartilhada, internalização, formalização e governança. Tais dimensões interagem e influenciam-se mutuamente. Todas fundamentam a CIP^(17)^.

As dimensões “Objetivos comuns” e “Visão compartilhada” relacionam-se com a interação entre os profissionais e a distribuição de responsabilidades. A dimensão “Internalização” está relacionada à consciência de interdependência com outros profissionais e à natureza do seu trabalho. Na “Formalização”, as regras são estabelecidas e relacionadas com as intervenções clínicas e as modalidades de interação são instituídas. E por fim, a dimensão da “Governança” diz respeito às organizações e seus fatores externos e internos de estrutura e poder^(17)^.

Diante do exposto, delineou-se o seguinte objetivo: analisar a atuação dos docentes e preceptores do Programa de Educação pelo Trabalho para a Saúde no desenvolvimento da CIP na Atenção Primária à Saúde.

## MÉTODO

### Desenho do Estudo

Pesquisa descritiva, de natureza qualitativa, que integra a tese intitulada “Colaboração Interprofissional no Programa de Educação pelo Trabalho para a Saúde/Interprofissionalidade”. Aplicou-se *checklist* do *Consolidated Criteria for Reporting Qualitative Research* (COREQ)^(18)^ e foram observados os requisitos relacionados aos métodos e aos demais tópicos, os quais serão discutidos a seguir.

### Local

Cinco universidades federais que desenvolvem atividades do PET-Saúde/Interprofissionalidade na APS do Nordeste do Brasil, as quais participaram do programa em duas edições anteriores (edital número 14/2013 – PET-Saúde/Redes de Atenção à Saúde e o edital número 13/2015 PET-Saúde/Gradua SUS) no mesmo *campus*.

### População

Docentes e preceptores do PET/Interprofissionalidade. O critério de inclusão estabelecido foi estar atuante no Programa por um período superior a 12 meses. Foram convidadas por e-mail (carta-convite) 130 pessoas, entre docentes e preceptores do PET/Interprofissionalidade. Destas, 60 aceitaram o convite e 44 responderam aos formulários, resultando assim em uma taxa de resposta de 73%. Para os que aceitaram o convite, foi enviado por e-mail o Termo de Consentimento Livre e Esclarecido (TCLE) e o *link* de acesso ao questionário. Estabeleceu-se o período de 30 dias como tempo médio entre o envio do convite e o recebimento das respostas on-line.

### Coleta De Dados

Na fase da coleta de dados, foram realizados seis testes-pilotos com preceptoras e docentes do PET-Saúde/Interprofissionalidade de universidades estaduais, seguindo o seguinte protocolo de realização: convite, envio TCLE com *link* de acesso do questionário, recebimento e análise das respostas, com comentários finais.

Após o aprimoramento do instrumento de coleta, foi realizada a coleta de dados por questionário aberto *on-line*, com instrução sobre o preenchimento, contento 14 questões, sendo elaborado e aplicado por uma pesquisadora experiente, por meio do *link* de acesso disponibilizado via *Google Forms*® entre os meses de agosto e outubro de 2020. O tempo de preenchimento para o questionário foi de 10 a 12 minutos.

Das questões formuladas, foram selecionadas aquelas relacionadas à caracterização dos participantes (sexo, graduação) e resposta sobre as estratégias utilizadas para introdução e ajustes à CIP. Não houve recusa ou suspensão da participação na pesquisa. Todos os participantes receberam uma cópia das respostas, o que permitiu confirmá-las.

### Análise e Tratamento de Dados

Os dados advindos do questionário via *Google Forms*® foram transportados para uma planilha de Excel e transpostos como fontes internas para o *software* webQDA®. Com auxílio desse *software*, as pesquisadoras construíram o *corpus* da pesquisa, realizaram as operações de codificação e análise dos dados, e estabeleceram os índices e a frequência absoluta das 50 palavras mais recorrentes relacionadas à CIP no PET-Saúde/Interprofissionalidade.

A árvore de codificação adotou o formato hierárquico, a partir da classificação das fontes em critérios nomeados a priori como: identificação e compartilhamento de objetivos comuns, internalização da interdependência de outro profissional de saúde e ajuste para o desenvolvimento da CIP.

A análise e o tratamento dos dados deram-se por análise de conteúdo^(19)^, ancorada no quadro teórico do Modelo de Estruturação da CIP^(17)^ que se baseia no princípio da CIP em contextos da Atenção Primária a Saúde e presente no processo de surgimento e estruturação, a partir da perspectiva das interações.

A categorização dos dados foi feita por agregação das unidades de registro pelo critério palavras-tema, ou seja, a partir de enunciado temático. Em seguida, foram feitas as inferências a partir dos dados.

### Aspectos Éticos

A pesquisa foi aprovada em 01 de julho de 2020 pelo Comitê de Ética em Pesquisa (CEP) da Escola de Enfermagem da Universidade Federal da Bahia, sob parecer de nº 4.127.223. Os compromissos éticos foram respeitados, conforme orientações do Conselho Nacional de Saúde, para procedimentos em pesquisas com seres humanos, em qualquer etapa e em ambiente virtual^(20)^. Para garantir o anonimato dos entrevistados, utilizou-se o codinome Entrevistado com a letra E, seguido do numeral ordinal apresentado por ordem crescente, a citar E1, E2 e assim sucessivamente. Conforme preconizado pela Ciência Aberta, foi disponibilizado o *link* do repositório no qual os dados de pesquisa foram depositados.

## RESULTADOS

O estudo foi realizado com 44 participantes (26 preceptores, 18 docentes do PET), a maioria do sexo feminino, com diferentes graduações em saúde, tais como Ciências Biológicas, Enfermagem, Fisioterapia, Medicina, Nutrição, Odontologia, Psicologia e Serviço Social. Desses, o maior percentual (45,5%) foi da Enfermagem.

Para melhor compreensão, e à luz do Modelo de Estruturação da CIP^(17)^, a atuação dos docentes e preceptores na condução da CIP está descrita em três das quatro dimensões expressas nos resultados, nas seguintes categorias estabelecidas: 1) Condução para o compartilhamento de objetivos comuns, 2) Mobilização para a internalização da interdependência entre profissionais e 3) Acompanhamento e ajuste para o desenvolvimento da colaboração interprofissional.

1) Condução para o Compartilhamento de Objetivos Comuns:

Durante as atividades realizadas no PET-Saúde/Interprofissionalidade, docentes e preceptores conduziram a inserção dos estudantes no serviço de saúde e no processo de trabalho da equipe de saúde.

*A inserção na realidade do serviço e do processo de trabalho da equipe de saúde, a partir do planejamento, execução e discussão das ações realizadas dentro e fora da Unidade Básica de Saúde* [...] *de forma articulada com os serviços e gestão* (E6).

[...] *Através do Diagnóstico Situacional levantado, possibilitou-se averiguar os possíveis problemas e entraves a serem trabalhados para a melhoria da assistência* (E12).

Dentre as ações desenvolvidas, destacaram-se as reuniões de equipe, nas quais motivaram a interação e o estabelecimento de objetivos em comum. Concomitantemente, houve estímulo à escuta de outros profissionais de saúde, bem como dos usuários e da comunidade, conforme apontado a seguir:

*Reunir todos os membros das equipes na qual os grupos tutoriais atuam, realizar oficinas, reuniões, debates para conhecimento sobre as profissões e práticas, de identificação dos pontos comuns das profissões, realizar planejamento de atividades na perspectiva interprofissional, estimular a escuta do usuário e a reflexão sobre a resposta do serviço às demandas dos usuários* [...] (E5).

*Encontros de definição de prioridades, planejamento e programação, avaliação, construção de agendas de atividades, organização e realização das atividades, estudos temáticos, tudo feito conjuntamente* (E39).

As respostas dos participantes revelaram que a interação dos sujeitos do Programa em pequenos grupos e o alinhamento conceitual constituem outras ações empreendidas pelos orientadores do PET-Saúde/Interprofissionalidade. Essas ações aconteceram no contexto pandêmico, vivenciado na época:

*Durante esse período* [...] *temos investido na qualificação dos estudantes, preceptores e docentes*, [...]. *As reuniões e encontros continuam acontecendo remotamente* (E6).

[...] *buscamos manter a organização de atividades em pequenos grupos* (preservando a composição multiprofissional) *e encontros regulares com todos os participantes do Grupo de Aprendizagem Tutorial para compartilhar experiências e debatermos aspectos conceituais*. [...] (E7).

2) Mobilização para a Internalização da interdependência entre profissionais:

Nesta categoria observou-se que as ações relacionadas à internalização da interdependência do outro partem de debates sobre as atribuições de cada profissão, discussão de casos, articulação centrada na comunidade e desenvolvimento do domínio das competências colaborativas, assim como descrito a seguir:

*Debates que promovam o conhecimento do papel de cada profissão, discussão de casos/situações problemáticas para identificação do papel de cada profissão na resolução do problema, com discussão das competências específicas, comuns e colaborativas* [...] (E7).

*A concepção pedagógica das ações do PET-Saúde/Interprofissionalidade das quais participo teve como intencionalidade dois princípios: atenção centrada na comunidade e foco no indivíduo e quatro domínios de competências: comunicação interprofissional, papéis profissionais, trabalho em equipe, ética e valores interprofissionais* (E18).

Ademais, os entrevistados demonstraram utilizar o fazer de modo conjunto, que pode acontecer durante a implementação de um projeto terapêutico, visita domiciliária ou execução de uma roda de conversa.

*As atividades são desenvolvidas nas Unidades Básicas de Saúde, junto aos profissionais da equipe e comunidade* [...] *Educação permanente, projeto terapêutico singular, consultas e visitas domiciliares compartilhadas* [...] (E8).

[...] *divisão de grupos de trabalho, respeitando a diversidade dos cursos e profissionais, acompanhamento da execução das atividades, rodas de conversa com integrantes de diferentes profissões* [...] (E26).

Segundo alguns participantes, as ações realizadas conjuntamente resultam em encadeamento de atividades estabelecidas para priorizar a necessidade de interdependência entre os participantes do Programa, usuários e equipe:

*Com a equipe, o foco era a elaboração de estratégias para o trabalho colaborativo; com os usuários, o foco era identificar demandas do cuidado em saúde para fortalecimento de ações interprofissionais* [...] (E11).

[...] *São traçadas as atividades a serem desenvolvidas com cada família e a meta a ser alcançada. As atividades são desenvolvidas, avaliadas e aplicadas. Em seguida, é levado para uma discussão em grupo* (alunos, preceptores e docentes) *e avaliado o sucesso ou não da atividade/atendimento. Assim, as famílias vão sendo atendidas de forma interprofissional. Para resolver problemáticas presentes nessas famílias* [...] (E25).

Em uma das entrevistas, foi feita menção a essa parceria por parte de um participante do PET-Saúde/Interprofissionalidade:

*Participação dos alunos, profissionais e comunidade nas práticas diárias do Serviço, com oportunidade de participação de todos nos processos de cuidado em saúde* [...] (E12).

[...] *parceria com redes organizadas de pacientes, reuniões com pacientes e seus médicos assistentes, estudos individuais e em grupo, pesquisas sobre os atendimentos com os usuários do serviço por caixa de sugestões* (E13).

3) Acompanhamento e ajuste para o desenvolvimento da colaboração interprofissional:

A terceira categoria está relacionada aos ajustes realizados por docentes e preceptoras do PET-Saúde/Interprofissionalidade para o desenvolvimento da CIP. Uma das ações foi a (re)condução do fazer colaborativo, por meio do diálogo e do *feedback* da comunidade:

*O diálogo é sempre o primeiro passo. Os grupos e reuniões semanais auxiliam muito na organização das atividades e no alinhamento das ações de cuidado e estratégias de interação e intervenção* (E22).

*Uma das demandas da comunidade era a implementação do grupo de mulheres. A cada ação realizada, era colhido o feedback da própria comunidade participante, com isso as próximas ações e participações podiam ser planejadas e ajustadas* [...] (E37).

Outro aspecto oriundo dos depoimentos diz respeito ao alinhamento das condutas e redefinição das rotas de atividades:

*Estudo de artigos científicos da Interprofissionalidade, reconhecimento da equipe multiprofissional e análise da consulta ampliada [...]. Construir projetos terapêuticos singulares (PTS) para alinhar condutas e encaminhamentos* (E3).

*Temos estimulado o debate sobre como tem sido realizado o trabalho em equipe, como tem sido a escuta entre os membros na realização das tarefas, quais estratégias eles têm utilizado na resolução de conflitos entre os membros e no equacionamento das decisões coletivas* [...] *para estimular a reflexão e a adoção de práticas solidárias e colaborativas* (E44).

No que se refere aos ajustes para o desenvolvimento da CIP, os achados evidenciam a mobilização intra e interinstitucional em alguns setores da saúde e da educação:

[...] *Envolvemos o* (setor de recursos humanos) *da Secretaria Municipal de Saúde em todas as etapas do projeto, mantendo-se cientes de todo planejamento e atividades realizadas; os professores pautam o tema da interprofissionalidade nas reuniões do Núcleo Docente Estruturante* [...] (E5).

[...] *Realização de encontros com os departamentos dos cursos de saúde, buscando sensibilizar os diversos atores da formação* (E41).

## DISCUSSÃO

É notória a representatividade das enfermeiras no que se refere à atuação dos docentes e preceptores do Programa de Educação pelo Trabalho para a Saúde no desenvolvimento da CIP na APS, coerente com a representatividade da profissão nos diversos cenários. Autores argumentam que as enfermeiras têm atitudes mais positivas para a colaboração interprofissional^(6)^.

Atuando como docentes ou preceptoras juntamente com outras categorias profissionais, as enfermeiras mobilizam ações para o compartilhamento de objetivos comuns, internalização da interdependência entre profissionais de saúde e acompanhamento e ajustes para o desenvolvimento da CIP. O conhecimento acerca das estratégias utilizadas pelas enfermeiras e demais profissionais de saúde evidencia a importância de investimento em experiências de aprendizado e cuidados interprofissionais, formação voltada para a experimentação, imersão e reflexão em contextos de trabalho em equipe de saúde da família e da comunidade.

No que diz respeito à condução para o compartilhamento de objetivos comuns - sendo este o ponto de partida em direção à CIP, ao inserirem os estudantes no cotidiano e processos de trabalho da equipe de saúde da família de modo interativo e em pequenos grupos, os facilitadores direcionam suas ações em convergência com o Modelo de Estruturação da CIP^(17)^. Ao promoverem a escuta entre profissionais, usuários e comunidade, os estudantes são estimulados a tomar decisões compartilhadas. Dessa forma, buscam produzir respostas frente à realidade encontrada, assim como verificado em outros estudos desenvolvidos em contextos semelhantes^(13,21)^. Contudo, embora o trabalho em equipe constitua uma realidade na APS^(22)^, ainda se percebe a valorização da consulta individual^(23)^.

No que se refere à mobilização para a internalização da interdependência entre profissionais de saúde, este estudo evidenciou que docentes e preceptoras do PET-Saúde/Interprofissionalidade têm disposição para investir na convivência mútua e na relação de confiança, elementos importantes na dimensão da Internalização da CIP. Os debates, as discussões de casos, as consultas e as visitas compartilhadas entre os participantes do PET-Saúde/Interprofissionalidade possibilitam saber sobre o outro profissional de saúde e influenciam as dimensões dos “Objetivos comuns” e das “Metas compartilhadas”, uma vez que se apropriam do cuidado colaborativo e do conhecimento do outro.

Quando os estudantes são estimulados a criar projetos terapêuticos, buscar articulação com pacientes e sua rede organizacional, potencializa-se o desenvolvimento da comunicação interprofissional e do cuidado centrado na pessoa, família e comunidade^(8)^. Desenvolver tais competências é imprescindível para o desenvolvimento da colaboração interprofissional^(24,25)^.

Embora se evidencie a potência de qualificar o cuidado em saúde por meio da atenção centrada no usuário, ainda é um desafio envolver os usuários na tomada de decisão sobre seus cuidados em saúde, conforme recomenda a Organização Mundial de Saúde^(26,27)^.

No acompanhamento e ajustes para o desenvolvimento da CIP, esta faz conexão com duas dimensões do Modelo de Estruturação da CIP: Formalização e Governança^(17)^. O alinhamento das condutas e a redefinição de rotas de atividades conduzidas pelos docentes e preceptores desse estudo alinham-se com a dimensão “Formalização”, uma vez que se apresentam sob a forma de troca de informações e formatação do processo de trabalho.

O diálogo entre os participantes do PET-Saúde/Interprofissionalidade e o *feedback* da comunidade na re(condução) do fazer colaborativo possibilitam valorizar e respeitar o usuário, esclarecer as responsabilidades e negociar como serão compartilhadas. Diante disso, é primordial superar as barreiras e desenvolver uma comunicação clara^(10)^, uma vez que, segundo a pesquisa, o comprometimento da sinergia entre a equipe e a dificuldade na comunicação constituem obstáculos à colaboração interprofissional^(4)^.

Outra ação identificada para o acompanhamento e o ajuste no desenvolvimento da CIP foi a mobilização intra e interinstitucional. Tal ação alinha-se com a dimensão “Governança” do Modelo de Estruturação da CIP. O papel político estratégico de docentes e preceptores do PET possibilitou uma direção clara para os estudantes, bem como um direcionamento para aprendizagem colaborativa.

Nos Estados Unidos da América, um programa de integração ensino-serviço semelhante ao PET-Saúde/Interprofissionalidade investiu na mobilização intra e interinstitucional para manutenção da CIP nos serviços de saúde^(8)^. Além da experiência brasileira do PET-Saúde/Interprofissionalidade, outros países como a África do Sul^(21)^ e Austrália^(28)^ apostaram na criação de disciplinas e estágios interprofissionais como espaços para o desenvolvimento da CIP.

As experiências de aprendizagem interprofissional focadas na internalização da interdependência do outro revelam-se significativas enquanto estimulam a mudança, tanto na formação, como na atuação dos profissionais de saúde. Esse tipo de aprendizagem condiz com a andragogia, educação para adultos, e contribui para o desenvolvimento de habilidades direcionadas à tomada de decisão^(29)^.

As limitações deste estudo residem na ausência de aprofundamento de questões relacionadas à influência dos fatores exógenos e estruturais, os quais interferem nos processos relacionais dos indivíduos. Assim, há necessidade de propostas investigativas sobre outros determinantes do processo colaborativo.

O avanço na área da saúde e da enfermagem também se relaciona com a mobilização continuada que o PET-Saúde/Interprofissionalidade tem para desenvolver práticas clínicas baseadas na colaboração e na interação entre profissionais, as quais permitem melhor integração para as práticas em saúde e contribuem para mudanças dos paradigmas para superar a fragmentação do cuidado e a supremacia do saber.

## CONCLUSÃO

Os resultados do estudo permitiram identificar as ações norteadas realizadas no PET-Saúde/Interprofissionalidade, sob a ótica dos docentes e preceptores. A atuação de docentes e preceptores no Programa favoreceu aproximações com a colaboração interprofissional, ao possibilitar ao estudante a introdução de novas práticas no âmbito relacional e a estruturação de ação coletiva entre a universidade e os serviços de saúde. O estudo permitiu ainda constatar que existe disposição dos facilitadores para conduzir um projeto educacional interprofissional. Todavia, seu trabalho esbarra na informalidade do processo de trabalho colaborativo, na baixa governabilidade das ações colaborativas e na fraca coesão entre a universidade e o serviço de saúde, em direção à implantação da colaboração interprofissional na saúde.

Diante dos contributos e das limitações, constata-se a necessidade de investir no compartilhamento de saberes entre os envolvidos, no respeito à liberdade de decisão e no envolvimento das famílias e da comunidade. Além disso, são necessárias novas pesquisas que avaliem a colaboração na efetividade das mudanças comportamentais dos profissionais de saúde, em diferentes espaços de integração entre ensino, serviço e comunidade.

## Data Availability

Todo o conjunto de dados que dá suporte aos resultados do estudo foi publicado no próprio artigo.

# Translation: Colaboración interprofesional en la atención primaria de salud: preceptores y docentes en la educación para el trabajo en salud

**DOI:** 10.1590/1980-220X-REEUSP-2024-0206es

**Authors:** Elaine Andrade Leal Silva, Rosana Maria de Oliveira Silva, Ana Lúcia Arcanjo Oliveira Cordeiro, Queuam Ferreira Silva de Oliveira, Rafaela Braga Pereira Velôso, Gilberto Tadeu Reis da Silva

**Affiliations:** 1 Universidade Federal do Recôncavo, Centro de Ciências da Saúde, Santo Antônio de Jesus, BA, Brasil.; 2 Universidade Federal da Bahia, Escola de Enfermagem, Salvador, BA, Brasil.; 3 Universidade do Estado da Bahia, Departamento de Educação, Senhor do Bonfim, BA, Brasil.; 4 Universidade Estadual de Feira de Santana, Departamento de Saúde, Feira de Santana, BA, Brasil.

**Keywords:** Educación Interprofesional, Equipo de Atención al Paciente, Preceptoría, Salud, Atención Primaria de Salud

## Abstract

**Objetivo:**

Este artículo tiene como objetivo analizar la actuación de docentes y preceptores del Programa de Educación por el Trabajo para la Salud en el desarrollo de la colaboración interprofesional en la Atención Primaria de Salud.

**Método:**

Estudio descriptivo, con abordaje cualitativo, en el que participaron docentes y preceptores del Programa de Educación por el Trabajo para la Salud de cinco universidades federales del Nordeste brasileño. Entre septiembre y octubre de 2020, 44 trabajadores respondieron al cuestionario en línea. Los datos fueron procesados de acuerdo con los procedimientos analíticos descritos por Bardin e interpretados a la luz del Modelo de Colaboración Interprofesional de cuatro dimensiones con la ayuda del software webQDA®.

**Resultados:**

Se identificaron tres categorías: 1) Conducir hacia el intercambio de objetivos comunes; 2) Movilizar hacia la internalización de la interdependencia entre profesionales; y 3) Monitorear y ajustar hacia el desarrollo de la colaboración interprofesional.

**Conclusión:**

El estudio reveló que el papel de los docentes y preceptores en el desarrollo de la colaboración interprofesional se centra significativamente en la conducta interactiva entre profesionales de la salud y estudiantes, así como en el desarrollo de acciones y ajustes para el trabajo colaborativo.

## INTRODUCCIÓN

La colaboración interprofesional en la Atención Primaria de Salud (APS) es un elemento esencial para el perfeccionamiento de la atención en salud, fomentando la integración de conocimientos y prácticas entre diferentes profesionales. Fundamentado en la Educación Interprofesional (EIP) y en la práctica interprofesional, este enfoque es un recurso estratégico para fortalecer el trabajo en equipo y potenciar los resultados en salud, especialmente enfrentando desafíos como las condiciones crónicas y la vulnerabilidad social.

Definida como una acción colectiva, la Colaboración Interprofesional (CIP) se basa en la asociación entre profesionales de la salud y usuarios, en el intercambio (planificación, intervención y responsabilidades), en la interdependencia (de uno hacia el otro) y en el poder (compartido entre los miembros del equipo)^(1)^. La adopción de estrategias que fomenten esta colaboración, como la educación a través del trabajo y la implementación de metodologías activas de enseñanza-aprendizaje, contribuye significativamente a la transformación de las prácticas y a la cualificación de la asistencia en la APS^(2)^.

La CIP se da en contextos de servicios de salud y comunitarios^(2,3)^ y, según la literatura, existen esfuerzos de las instituciones educativas en programas de educación interprofesional, reflexiones frente a las experiencias exitosas en el contexto de la APS, con el fin de preparar a los futuros profesionales de la salud para el desarrollo de la práctica interprofesional^(4,5)^. Un estudio realizado en Singapur con médicos y enfermeros concluyó que la CIP mejora los resultados de las personas atendidas, así como la satisfacción de los profesionales y los costes en salud^(6)^. Las investigaciones en Europa y Norteamérica avanzan en la difusión de procesos innovadores de formación en salud orientados a las necesidades de salud del usuario^(7,8)^.

Las investigaciones realizadas en Brasil anuncian un apoyo institucional limitado^(9,10)^ a la enseñanza y la práctica interprofesional, las intervenciones en equipo^(11,12)^, la flexibilidad en el plan de estudios, la inclusión de experiencias interprofesionales^(7,9)^, la articulación entre la enseñanza y el servicio^(9)^ y el desempeño profesional que se aleje de la práctica mecanizada y fragmentada^(10)^. Otras investigaciones, a partir de las acciones desarrolladas en el Programa de Educación por el Trabajo para la Salud (PET-Saúde), destacan el desarrollo de actividades colaborativas, en las que estudiantes, docentes y profesionales de la salud, juntos, de manera interactiva e interdependiente, compartieron aprendizajes e intercambiaron conocimientos entre diferentes cursos y profesiones^(11,13)^.

Sin embargo, en la práctica, todavía existe la fragmentación de la atención en salud, además de percepciones distorsionadas sobre las profesiones^(12)^. Invertir en el desarrollo de los estudiantes y profesionales de la salud^(4)^, así como en la acogida, el trabajo en equipo^(5)^ y las prácticas de atención sanitaria más colaborativas y comunitarias^(10,12)^, puede contribuir a la reorientación de la formación y la actuación en el ámbito de la salud.

Tal y como se propone en el programa mencionado, las universidades participantes desarrollan sus acciones basándose en los fundamentos teóricos y metodológicos de la EIP^(14,15)^ con el fin de fomentar la interprofesionalidad, así como formar profesionales aptos para el trabajo colaborativo. Dicho esto, se considera que el PET-Saúde/Interprofissionalidade (Programa de Educación por el Trabajo/Interprofesionalidad) es un espacio de interacción entre profesionales de la salud que estimula cambios curriculares alineados con las directrices curriculares de los cursos de salud e induce el trabajo multi e interprofesional en los escenarios de enseñanza-servicio^(16)^. Sin embargo, persisten obstáculos para el avance de la EIP en las universidades brasileñas^(10)^, lo que requiere la continuidad de la inversión en la formación en salud y la movilización de la investigación sobre la actuación de los educadores en la conducción de la CIP en el PET-Saúde/Interprofissionalidade.

Cabe destacar la importancia del protagonismo de los profesores y preceptores en la planificación, conducción y ejecución de la CIP desarrollada en el Programa, así como en las dimensiones del modelo tetradimensional elaborado por D’Amour y colaboradores^(17)^. Conocer cómo se está desarrollando el proceso formativo en programas inductores de la EIP, basado en un modelo que analiza las dimensiones relacionales y organizativas, puede contribuir a la evaluación y la implementación del Programa y de los planes de estudio interprofesionales.

Este modelo se estructura en cuatro dimensiones: objetivos comunes y visión compartida, internalización, formalización y gobernanza. Estas dimensiones interactúan y se influyen mutuamente. Todas ellas fundamentan la CIP^(17)^.

Las dimensiones «Objetivos comunes» y «Visión compartida» se relacionan con la interacción entre los profesionales y la distribución de responsabilidades. La dimensión «Internalización» está relacionada con la conciencia de interdependencia con otros profesionales y la naturaleza de su trabajo. En la «Formalización», se establecen las normas y se relacionan con las intervenciones clínicas y se instituyen las modalidades de interacción. Por último, la dimensión «Gobernanza» se refiere a las organizaciones y sus factores externos e internos de estructura y poder^(17)^.

En vista de lo anterior, se ha definido el siguiente objetivo: analizar la actuación de los docentes y preceptores del Programa de Educación para la Salud a través del Trabajo en el desarrollo de la CIP en la Atención Primaria de Salud.

## MÉTODO

### Diseño del Estudio

Investigación descriptiva, de naturaleza cualitativa, que forma parte de la tesis titulada «Colaboración interprofesional en el Programa de Educación para la Salud a través del Trabajo/Interprofesionalidad». Se aplicó la *lista de verificación* de los *Criterios Consolidados para la Presentación de Informes de Investigación Cualitativa* (COREQ)^(18)^ y se observaron los requisitos relacionados con los métodos y otros temas, que se discutirán a continuación.

### Lugar del Estudio

Cinco universidades federales que desarrollan actividades del PET-Saúde/Interprofesionalidad en la APS del Nordeste de Brasil, las cuales participaron en el programa en dos ediciones anteriores (convocatoria número 14/2013 – PET-Saúde/Redes de Atención a la Salud y convocatoria número 13/2015 PET-Saúde/Gradua SUS) en el mismo campus.

### Población

Docentes y preceptores del PET/Interprofesionalidad. El criterio de inclusión establecido fue haber participado en el Programa durante un período superior a 12 meses.

Se invitó por correo electrónico (carta de invitación) a 130 personas, entre docentes y preceptores del PET/Interprofesionalidad. De ellos, 60 aceptaron la invitación y 44 respondieron a los formularios, lo que supuso una tasa de respuesta del 73%. A quienes aceptaron la invitación se les envió por correo electrónico el Término de Consentimiento Libre y Esclarecido (TCLE) y el enlace de acceso al cuestionario. Se estableció un período de 30 días como tiempo medio entre el envío de la invitación y la recepción de las respuestas en línea.

### Recolección de Datos

En la fase de recolección de datos, se realizaron seis pruebas piloto con preceptoras y docentes del PET-Saúde/Interprofissionalidade de universidades estatales, siguiendo el siguiente protocolo de realización: invitación, envío del TCLE con *enlace* de acceso al cuestionario, recepción y análisis de las respuestas, con comentarios finales.

Después de perfeccionar el instrumento de recolección, se llevó a cabo la recolección de datos mediante un cuestionario abierto en línea, con instrucciones para su llenado, que contenía 14 preguntas, elaborado y aplicado por una investigadora con experiencia, a través del enlace de acceso facilitado a través de *Google Forms*® entre los meses de agosto y octubre de 2020. El tiempo de llenado del cuestionario fue de 10 a 12 minutos.

De las preguntas formuladas, se seleccionaron aquellas relacionadas con la caracterización de los participantes (sexo, titulación) y la respuesta sobre las estrategias utilizadas para la introducción y los ajustes a la CIP. No hubo rechazo ni suspensión de la participación en la investigación. Todos los participantes recibieron una copia de las respuestas, lo que permitió confirmarlas.

### Análisis y Tratamiento de Datos

Los datos obtenidos del cuestionario a través de *Google Forms*® se transfirieron a una hoja de cálculo de Excel y se transpusieron como fuentes internas al software webQDA®. Con la ayuda de este software, las investigadoras construyeron el *corpus* de la investigación, realizaron las operaciones de codificación y análisis de los datos, y establecieron los índices y la frecuencia absoluta de las 50 palabras más recurrentes relacionadas con la CIP en el PET-Saúde/Interprofissionalidade.

El árbol de codificación adoptó un formato jerárquico, a partir de la clasificación de las fuentes en criterios nombrados a priori como: identificación y compartir objetivos comunes, internalización de la interdependencia de otro profesional de la salud y ajuste para el desarrollo de la CIP.

El análisis y el tratamiento de los datos se realizaron mediante análisis de contenido^(19)^, basado en el marco teórico del Modelo de Estructuración de la CIP^(17)^, que se basa en el principio de la CIP en contextos de Atención Primaria de Salud y presente en el proceso de surgimiento y estructuración, desde la perspectiva de las interacciones.

La categorización de los datos se realizó mediante la agregación de las unidades de registro por el criterio de palabras-tema, es decir, a partir de enunciados temáticos. A continuación, se realizaron las inferencias a partir de los datos.

### Aspectos Éticos

La investigación fue aprobada el 1 de julio de 2020 por el Comité de Ética en Investigación de la Escuela de Enfermería de la Universidad Federal de Bahía, bajo el dictamen n.º 4.127.223. Se respetaron los compromisos éticos, de acuerdo con las directrices del Consejo Nacional de Salud, para procedimientos en investigaciones con seres humanos, en cualquier etapa y en entorno virtual^(20)^. Para garantizar el anonimato de los entrevistados, se utilizó el nombre en clave Entrevistado con la letra E, seguida del número ordinal presentado en orden ascendente, citando E1, E2 y así sucesivamente. De acuerdo con lo recomendado por la Ciencia Abierta, se ha facilitado el *enlace* al repositorio en el que se han depositado los datos de la investigación.

## RESULTADOS

El estudio se realizó con 44 participantes (26 preceptores, 18 docentes del PET), en su mayoría mujeres, con diferentes titulaciones en salud, tales como Ciencias Biológicas, Enfermería, Fisioterapia, Medicina, Nutrición, Odontología, Psicología y Trabajo Social. De ellos, el mayor porcentaje (45,5%) era de Enfermería.

Para una mejor comprensión, y a la luz del Modelo de Estructuración de la CIP^(17)^, la actuación de los docentes y preceptores en la conducción de la CIP se describe en tres de las cuatro dimensiones expresadas en los resultados, en las siguientes categorías establecidas: 1) Conducción para el intercambio de objetivos comunes, 2) Movilización para la internalización de la interdependencia entre profesionales y 3) Acompañamiento y ajuste para el desarrollo de la colaboración interprofesional.

1) Conducción para compartir objetivos comunes:

Durante las actividades realizadas en el PET-Saúde/Interprofissionalidade, docentes y preceptores condujeron la inserción de los estudiantes en el servicio de salud y en el proceso de trabajo del equipo de salud.

*La inserción en la realidad del servicio y del proceso de trabajo del equipo de salud, a partir de la planificación, ejecución y discusión de las acciones realizadas dentro y fuera de la Unidad Básica de Salud* [...] *de forma articulada con los servicios y la gestión* (E6).

[...] *A través del diagnóstico situacional realizado, se pudieron identificar los posibles problemas y obstáculos que debían abordarse para mejorar la asistencia* (E12).

Entre las acciones desarrolladas, destacaron las reuniones de equipo, en las que se motivó la interacción y el establecimiento de objetivos comunes. Al mismo tiempo, se estimuló la escucha de otros profesionales de la salud, así como de los usuarios y la comunidad, tal y como se señala a continuación:

*Reunir a todos los miembros de los equipos en los que actúan los grupos tutoriales, realizar talleres, reuniones, debates para conocer las profesiones y prácticas, identificar los puntos comunes de las profesiones, planificar actividades desde una perspectiva interprofesional, estimular la escucha del usuario y la reflexión sobre la respuesta del servicio a las demandas de los usuarios* [...] (E5).

*Reuniones para definir prioridades, planificar y programar, evaluar, elaborar agendas de actividades, organizar y llevar a cabo las actividades, realizar estudios temáticos, todo ello de forma conjunta* (E39).

Las respuestas de los participantes revelaron que la interacción de los sujetos del Programa en pequeños grupos y la alineación conceptual constituyen otras acciones emprendidas por los orientadores del PET-Saúde/Interprofesionalidad. Estas acciones tuvieron lugar en el contexto pandémico que se vivía en ese momento:

*Durante este período* [...] *hemos invertido en la cualificación de los estudiantes, preceptores y docentes,* [...]. *Las reuniones y encuentros siguen celebrándose de forma remota* (E6).

[...] *buscamos mantener la organización de actividades en pequeños grupos* (preservando la composición multiprofesional) *y encuentros regulares con todos los participantes del Grupo de Aprendizaje Tutorial para compartir experiencias y debatir aspectos conceptuales.* [...] (E7).

2) Movilización para la internalización de la interdependencia entre profesionales:

En esta categoría se observó que las acciones relacionadas con la internalización de la interdependencia del otro parten de debates sobre las atribuciones de cada profesión, la discusión de casos, la articulación centrada en la comunidad y el desarrollo del dominio de las competencias colaborativas, tal y como se describe a continuación:

*Debates que promuevan el conocimiento del papel de cada profesión, discusión de casos/situaciones problemáticas para identificar el papel de cada profesión en la resolución del problema, con discusión de las competencias específicas, comunes y colaborativas* [...] (E7).

*La concepción pedagógica de las acciones del PET-Saúde/Interprofissionalidade en las que participo tenía como intencionalidad dos principios: atención centrada en la comunidad y enfoque en el individuo y cuatro ámbitos de competencias: comunicación interprofesional, roles profesionales, trabajo en equipo, ética y valores interprofesionales* (E18).

Además, los entrevistados demostraron utilizar el hacer de manera conjunta, lo que puede ocurrir durante la implementación de un proyecto terapéutico, una visita domiciliaria o la realización de una ronda de conversación.

*Las actividades se desarrollan en las Unidades Básicas de Salud, junto con los profesionales del equipo y la comunidad* [...] *Educación permanente, proyecto terapéutico singular, consultas y visitas domiciliarias compartidas* [...] (E8).

[...] *división de grupos de trabajo, respetando la diversidad de cursos y profesionales, seguimiento de la ejecución de las actividades, rondas de conversación con miembros de diferentes profesiones* [...] (E26).

Según algunos participantes, las acciones realizadas conjuntamente dan lugar a una cadena de actividades establecidas para priorizar la necesidad de interdependencia entre los participantes del Programa, los usuarios y el equipo:

*Con el equipo, el enfoque era la elaboración de estrategias para el trabajo colaborativo; con los usuarios, el enfoque era identificar las demandas de atención en salud para fortalecer las acciones interprofesionales* [...] (E11).

[...] *Se trazan las actividades que se desarrollarán con cada familia y la meta a alcanzar. Las actividades se desarrollan, evalúan y aplican. A continuación, se lleva a un debate en grupo (alumnos, preceptores y docentes) y se evalúa el* éxito *o el fracaso de la actividad/atención. De este modo, las familias reciben una atención interprofesional. Para resolver los problemas presentes en estas familias* [...] (E25).

En una de las entrevistas, un participante del PET-Saúde/Interprofissionalidade mencionó esta colaboración:

*Participación de los alumnos, los profesionales y la comunidad en las prácticas diarias del Servicio, con oportunidad de participación de todos en los procesos de atención en salud* [...] (E12).

[...] *colaboración con redes organizadas de pacientes, reuniones con pacientes y sus médicos asistentes, estudios individuales y en grupo, investigaciones sobre la atención a los usuarios del servicio mediante un buzón de sugerencias* (E13).

3) Seguimiento y ajuste para el desarrollo de la colaboración interprofesional:

La tercera categoría está relacionada con los ajustes realizados por los docentes y preceptores del PET-Saúde/Interprofissionalidade para el desarrollo de la CIP. Una de las acciones fue la (re)orientación del trabajo colaborativo, a través del diálogo y el *feedback* de la comunidad:

*El diálogo es siempre el primer paso. Los grupos y las reuniones semanales ayudan mucho a organizar las actividades y a alinear las acciones de atención y las estrategias de interacción e intervención* (E22).

*Una de las demandas de la comunidad era la implementación del grupo de mujeres. Después de cada acción realizada, se recababa la retroalimentación de la propia comunidad participante, con lo que se podían planificar y ajustar las próximas acciones y participaciones* [...] (E37).

Otro aspecto que se desprende de los testimonios se refiere a la armonización de las conductas y la redefinición del camino de las actividades:

*Estudio de artículos científicos sobre interprofesionalidad, reconocimiento del equipo multiprofesional y análisis de la consulta ampliada* [...]. *Elaborar proyectos terapéuticos singulares (PTS) para armonizar conductas y derivaciones* (E3).

*Hemos estimulado el debate sobre cómo se ha llevado a cabo el trabajo en equipo, cómo ha sido la escucha entre los miembros en la realización de las tareas, qué estrategias han utilizado en la resolución de conflictos entre los miembros y en la toma de decisiones colectivas* [...] *para estimular la reflexión y la adopción de prácticas solidarias y colaborativas (E44).*

En lo que respecta a los ajustes para el desarrollo de la CIP, los hallazgos evidencian la movilización intra e interinstitucional en algunos sectores de la salud y la educación:

[...] *Involucramos al* (sector de recursos humanos) *de la Secretaría Municipal de Salud en todas las etapas del proyecto, manteniéndolo al tanto de toda la planificación y las actividades realizadas; los profesores incluyen el tema de la interprofesionalidad en las reuniones del Núcleo Docente Estructurante* [...] (E5).

[...] *Realización de reuniones con los departamentos de los cursos de salud, con el fin de sensibilizar a los diferentes actores de la formación* (E41).

## DISCUSIÓN

Es notable la representatividad de las enfermeras en lo que se refiere a la actuación de los docentes y preceptores del Programa de Educación por el Trabajo para la Salud en el desarrollo de la CIP en la APS, en consonancia con la representatividad de la profesión en los diferentes escenarios. Los autores argumentan que las enfermeras tienen actitudes más positivas hacia la colaboración interprofesional^(6)^.

Actuando como docentes o preceptoras junto con otras categorías profesionales, las enfermeras movilizan acciones para compartir objetivos comunes, internalizar la interdependencia entre los profesionales de la salud y realizar un seguimiento y ajustes para el desarrollo de la CIP. El conocimiento sobre las estrategias utilizadas por las enfermeras y otros profesionales de la salud evidencia la importancia de invertir en experiencias de aprendizaje y cuidados interprofesionales, formación orientada a la experimentación, la inmersión y la reflexión en contextos de trabajo en equipo de salud familiar y comunitaria.

En lo que respecta a la conducción hacia el intercambio de objetivos comunes, que es el punto de partida hacia la CIP, al insertar a los estudiantes en el cotidiano y los procesos de trabajo del equipo de salud familiar de manera interactiva y en pequeños grupos, los facilitadores dirigen sus acciones en convergencia con el Modelo de Estructuración de la CIP^(17)^. Al promover la escucha entre profesionales, usuarios y comunidad, se estimula a los estudiantes a tomar decisiones compartidas. De esta forma, buscan producir respuestas frente a la realidad encontrada, tal como se ha verificado en otros estudios desarrollados en contextos similares^(13,21)^. Sin embargo, aunque el trabajo en equipo es una realidad en la APS^(22)^, todavía se percibe la valoración de la consulta individual^(23)^.

En lo que se refiere a la movilización para la internalización de la interdependencia entre los profesionales de la salud, este estudio puso de manifiesto que los docentes y preceptores del PET-Saúde/Interprofissionalidade están dispuestos a invertir en la convivencia mutua y en la relación de confianza, elementos importantes en la dimensión de la Internalización de la CIP. Los debates, las discusiones de casos, las consultas y las visitas compartidas entre los participantes del PET-Saúde/Interprofissionalidade permiten conocer al otro profesional de la salud e influyen en las dimensiones de los «Objetivos comunes» y las «Metas compartidas», ya que se apropian del cuidado colaborativo y del conocimiento del otro.

Cuando se estimula a los estudiantes a crear proyectos terapéuticos, a buscar la articulación con los pacientes y su red organizativa, se potencia el desarrollo de la comunicación interprofesional y la atención centrada en la persona, la familia y la comunidad^(8)^. El desarrollo de estas competencias es imprescindible para el desarrollo de la colaboración interprofesional^(24,25)^.

Aunque es evidente el potencial de mejorar la atención sanitaria mediante la atención centrada en el usuario, sigue siendo un reto involucrar a los usuarios en la toma de decisiones sobre su atención en salud, tal y como recomienda la Organización Mundial de la Salud^(26,27)^.

En el seguimiento y los ajustes para el desarrollo de la CIP, esta se conecta con dos dimensiones del Modelo de Estructuración de la CIP: Formalización y Gobernanza^(17)^. La alineación de las conductas y la redefinición de las rutas de las actividades realizadas por los docentes y preceptores de este estudio se alinean con la dimensión «Formalización», ya que se presentan en forma de intercambio de información y formateo del proceso de trabajo.

El diálogo entre los participantes del PET-Saúde/Interprofissionalidade y el *feedback* de la comunidad en la (re)conducción del hacer colaborativo permiten valorar y respetar al usuario, aclarar las responsabilidades y negociar cómo se compartirán. Ante esto, es fundamental superar las barreras y desarrollar una comunicación clara^(10)^, ya que, según la investigación, el compromiso de la sinergia entre el equipo y la dificultad en la comunicación constituyen obstáculos para la colaboración interprofesional^(4)^.

Otra acción identificada para el seguimiento y el ajuste en el desarrollo de la CIP fue la movilización intra e interinstitucional. Esta acción se alinea con la dimensión «Gobernanza» del Modelo de Estructuración de la CIP. El papel político estratégico de los docentes y preceptores del PET permitió una orientación clara para los estudiantes, así como una orientación hacia el aprendizaje colaborativo.

En los Estados Unidos de América, un programa de integración enseñanza-servicio similar al PET-Saúde/Interprofissionalidade invirtió en la movilización intra e interinstitucional para el mantenimiento de la CIP en los servicios de salud^(8)^. Además de la experiencia brasileña del PET-Saúde/Interprofissionalidade, otros países como Sudáfrica^(21)^ y Australia^(28)^ apostaron por la creación de disciplinas y prácticas interprofesionales como espacios para el desarrollo de la CIP.

Las experiencias de aprendizaje interprofesional centradas en la internalización de la interdependencia del otro resultan significativas, ya que estimulan el cambio, tanto en la formación como en la actuación de los profesionales de la salud. Este tipo de aprendizaje es coherente con la andragogía, la educación para adultos, y contribuye al desarrollo de habilidades orientadas a la toma de decisiones^(29)^.

Las limitaciones de este estudio residen en la falta de profundización en cuestiones relacionadas con la influencia de factores exógenos y estructurales, que interfieren en los procesos relacionales de los individuos. Por lo tanto, es necesario investigar otros determinantes del proceso colaborativo.

El avance en el área de la salud y la enfermería también se relaciona con la movilización continua que el PET-Saúde/Interprofissionalidade tiene para desarrollar prácticas clínicas basadas en la colaboración y la interacción entre profesionales, las cuales permiten una mejor integración de las prácticas en salud y contribuyen a cambios de paradigmas para superar la fragmentación de la atención y la supremacía del saber.

## CONCLUSIÓN

Los resultados del estudio permitieron identificar las acciones orientadas realizadas en el PET-Saúde/Interprofissionalidade, desde la perspectiva de los docentes y preceptores. La actuación de los docentes y preceptores en el Programa favoreció el acercamiento a la colaboración interprofesional, al permitir a los estudiantes introducir nuevas prácticas en el ámbito relacional y estructurar la acción colectiva entre la universidad y los servicios de salud. El estudio también permitió constatar que existe disposición por parte de los facilitadores para llevar a cabo un proyecto educativo interprofesional. Sin embargo, su trabajo se topa con la informalidad del proceso de trabajo colaborativo, la baja gobernabilidad de las acciones colaborativas y la débil cohesión entre la universidad y el servicio de salud, en el sentido de la implantación de la colaboración interprofesional en salud.

Ante las contribuciones y limitaciones, se constata la necesidad de invertir en el intercambio de conocimientos entre los involucrados, en el respeto a la libertad de decisión y en la participación de las familias y la comunidad. Además, se necesitan nuevas investigaciones que evalúen la colaboración en la efectividad de los cambios de comportamiento de los profesionales de la salud, en diferentes espacios de integración entre la enseñanza, el servicio y la comunidad.

## Data Availability

Todo el conjunto de datos que respalda los resultados del estudio fue publicado en el propio artículo.
